# Dual targeting of l-carnitine-conjugated nanoparticles to OCTN2 and ATB^0,+^ to deliver chemotherapeutic agents for colon cancer therapy

**DOI:** 10.1080/10717544.2017.1377316

**Published:** 2017-09-15

**Authors:** Longfa Kou, Qing Yao, Sathish Sivaprakasam, Qiuhua Luo, Yinghua Sun, Qiang Fu, Zhonggui He, Jin Sun, Vadivel Ganapathy

**Affiliations:** a Municipal Key Laboratory of Biopharmaceutics, Wuya College of Innovation, Shenyang Pharmaceutical University, Shenyang, China;; b Department of Cell Biology and Biochemistry, Texas Tech University Health Sciences Center, Lubbock, TX, USA;; c Department of Pharmaceutics, Wuya College of Innovation, Shenyang Pharmaceutical University, Shenyang, China

**Keywords:** l-Carnitine, nanoparticles, OCTN2, ATB^0,+^, colon cancer

## Abstract

l-Carnitine, obligatory for oxidation of fatty acids, is transported into cells by the Na^+^-coupled transporter OCTN2 and the Na^+^/Cl^–^-coupled transporter ATB^0,+^. Here we investigated the potential of L-carnitine-conjugated poly(lactic-co-glycolic acid) (PLGA) nanoparticles (LC-PLGA NPs) to deliver chemotherapeutic drugs into cancer cells by targeting the nanoparticles to both OCTN2 and ATB^0,+^. The cellular uptake of LC-PLGA NPs in the breast cancer cell line MCF7 and the colon cancer cell line Caco-2 was increased compared to unmodified nanoparticles, but decreased in the absence of co-transporting ions (Na^+^ and/or Cl^–^) or in the presence of competitive substrates for the two transporters. Studies with fluorescently labeled nanoparticles showed their colocalization with both OCTN2 and ATB^0,+^, confirming the involvement of both transporters in the cellular uptake of LC-PLGA NPs. As the expression levels of OCTN2 and ATB^0,+^ are higher in colon cancer cells than in normal colon cells, LC-PLGA NPs can be used to deliver chemotherapeutic drugs selectively into cancer cells for colon cancer therapy. With 5-fluorouracil-loaded LC-PLGA NPs, we were able to demonstrate significant increases in the uptake efficiency and cytotoxicity in colon cancer cells that were positive for OCTN2 and ATB^0,+^. In a 3D spheroid model of tumor growth, LC-PLGA NPs showed increased uptake and enhanced antitumor efficacy. These findings indicate that dual-targeting LC-PLGA NPs to OCTN2 and ATB^0,+^ has great potential to deliver chemotherapeutic drugs for colon cancer therapy.

Dual targeting LC-PLGA NPs to OCTN2 and ATB0,+ can selectively deliver chemotherapeutics to colon cancer cells where both transporters are overexpressed, preventing targeting to normal cells and thus avoiding off-target side effects.

## Introduction

Carnitine (β-hydroxy-γ-trimethylaminobutyrate), a high polar zwitterionic compound, facilitates the transport of long-chain fatty acid across the inner mitochondrial membrane for subsequent β-oxidation. It is critical for the tissues such as skeletal muscle and heart, which depend on fatty acid oxidation as the primary source of energy. Carnitine is synthesised endogenously by liver, kidney and brain, and is also absorbed from diet in intestinal tract (Rebouche & Seim, 1998). Transfer of carnitine across the plasma membrane in mammalian cells occurs primarily via the novel organic cation/carnitine transporter 2 (OCTN2, SLC22A5) (Tamai, 2013). OCTN2 is a Na^+^-dependent, high-affinity transporter for carnitine with an apparent K_m_ about 10 µM (Pochini et al., 2013). The neutral and basic amino acid transporter B(0+) (ATB^0,+^, SLC6A14) is also capable of carnitine transport, but it has relatively lower affinity than OCTN2, with an apparent K_m_ about 800 µM (Nakanishi et al., 2001; Hatanaka et al., 2004). ATB^0,+^ is a Na^+^- and Cl^–^-coupled transporter and mostly expressed in colon, lung and mammary gland (Bhutia et al., 2014).

Over the past few decades, significant progress has been made in the application of nanoparticles (liposomes, polymeric nanoparticles, micelles, dendrimers, etc.) for drug delivery (Couvreur, 2013; Sun et al., 2015). These nanoparticles have proved to be one of the most promising strategies for drug delivery due to the increased solubility and stability of drugs, optimal pharmacokinetics, and targeting. Targeted nanoparticles could take advantages of cell type-specific expressed macromolecules (antigens, receptors, or transporters) that are overexpressed on the cell surface. In combination with cell/tissue-specific release of encapsulated drugs, targeted nanoparticles could improve therapeutic index (Lian et al., 2013; Dai et al., 2016; Luo et al., 2016). Many promising advances have been achieved for the treatment of ovarian (Vilos et al., 2013; Pi et al., 2017), breast (Li et al., 2015; Campos et al., 2017), prostate (Hoang et al., 2014), and lung cancers (Chandolu & Dass, 2013; Karra et al., 2013), and a number of such systems are being in the clinical development (Kamaly et al., 2012). Recently, we have examined l-carnitine-conjugated poly(lactic-co-glycolic acid) nanoparticles (LC-PLGA NPs) for their utility for drug delivery (Kou et al., 2017). We used l-carnitine as the ligand on the surface of nanoparticles so that they could interact with OCTN2 expressed on luminal membrane of enterocytes to facilitate oral drug delivery. As l-carnitine is also a substrate of ATB^0,+^, we extended in the present study the utility of LC-PLGA NPs to target both OCTN2 and ATB^0,+^ using appropriate cell lines. As both these transporters are expressed at high levels in certain cancers (colon cancer, pancreatic cancer, ER-positive breast cancer), dual targeting of LC-PLGA NPs to these transporters would potentially be very effective for the delivery of chemotherapeutic agents in the treatment of such cancers.

Colon cancer is one of the most widely prevalent cancer worldwide with significant morbidity and mortality (Chen et al., 2016; Siegel et al., 2016). Surgery is the primary form of treatment for colon cancer. Recurrence following surgery and metastasis are major problems and could decrease the patients’ survival rate to 6% (Compton et al., 2012). Adjuvant chemotherapy is usually used after surgery to decrease the risk of recurrence and improve life expectancy. Nevertheless, the low specificity of chemotherapy agents often produces side effects including hair loss, nausea, and vomiting (Chabner & Roberts, 2005). The development of therapeutic strategies for cancer based on nanoparticles has undergone significant advances in recent years with the evidence of decreasing the nonspecific side effects and increasing therapeutic efficiency. Nanoparticles have been shown to be effective as a drug delivery system in the treatment of prostate (Nagesh et al., 2016; Sanna et al., 2017), lung (Ishiguro et al., 2016; Perepelyuk et al., 2016), breast (Bowerman et al., 2017; Fasehee et al., 2016), and ovarian (Cocco et al., 2016; Roberts et al., 2017) cancers. However, even though the high morbidity and mortality are associated with colon cancer, the clinical application of nanoparticles remains limited.

In the present study, we have found that both OCTN2 and ATB^0,+^ are expressed a much higher level in human colon cancer cells than in normal colon cells, which provides a unique opportunity to examine the potential dual targeting of LC-PLGA NPs to OCTN2 and ATB^0,+^ to enhance the delivery of chemotherapeutic agents in a tumor cell-specific manner. Our studies show that LC-PLGA NPs are taken up preferentially by colon cancer cells compared with normal colon epithelial cells and that both OCTN and ATB^0,+^ are involved in the process.

## Materials and methods

### Materials

5-Fluorouracil (5-FU), coumarin 6 and 3-(4, 5-dimethylthiazol-2-yl)-2, 5-diphenyltetrazolium bromide were purchased from Sigma-Adrich (St. Louis, MO). PLGA (38,000 mw) was purchased from Jinan Daigang Biological Engineering Co. Ltd. (Jinan, China). Poly(vinylalcohol) (20,000–30,000 mw) was purchased from Acros Organics (New Jersey, USA). Stearoyl-l-carnitine was prepared in our laboratory as reported recently (Kou et al., 2017). Antibodies against OCTN2 (SLC22A5) and ATB^0,+^ (SLC6A14) were obtained from Abcam (Cambridge, MA), and β-actin antibody was from Sigma-Aldrich (St. Louis, MO). Goat anti-rabbit IgG (H + L) labeled with Alexa Fluor^®^ 594, goat anti-rabbit IgG-HRP, and goat anti-mouse IgG-HRP were purchased from Santa Cruz Biotechnology, Inc. (Dallas, TX). All other chemicals and reagents were of analytical grade.

### Cell lines

All the cell lines used here were obtained from American Type Culture Collection (USA). MB231, CCD841, Caco-2, and LS174T were cultured in RPMI-1640 medium with 50 units/mL streptomycin, 100 units/mL penicillin and FBS (10%). MCF7 was cultured in DMEM medium with 50 units/mL streptomycin, 100 units/mL penicillin and FBS (10%). HCT116 and HT29 were cultured in McCoy’s 5 A medium with 50 units/mL streptomycin, 100 units/mL penicillin and FBS (10%). All cells were cultured at 37 °C in a humidified incubator with 5% CO2.

### Preparation and characterization of LC-PLGA NPs

The methods of preparation and characterization of LC-PLGA NPs with various ligand densities were displayed in supporting information.

### Analysis of OCTN2 and ATB^0,+^ expression in cells

Cells were harvested and lysed in radioimmuno-precipitation assay buffer (RIPA buffer) supplemented with protease and phosphatase inhibitor cocktail and 0.01 M EDTA solution. BCA method was used to measure the total amount of proteins. Proteins extracts (20 μg) were subjected to electrophoresis in SDS-PAGE on a 12% gel, separated and transferred to nitrocellulose membranes (0.2 μm). The membrane was blocked with 5% nonfat milk in TBST (10 mM Tris-HCl, 150 mM NaCl, and 0.05% Tween-20) for 1 h and incubated with primary antibodies against OCTN2, ATB^0,+^ and β-actin overnight at 4 °C. After being washed three times for 10 min each with TBST, the membranes were incubated with HRP-conjugated secondary antibody. After another 3-time wash with TBST, the membranes were incubated with ECL substrate for 5 min, then the membranes were developed using X-ray machine.

### Uptake assay

Cells were seeded in 24-well plates at a density of 1.5 × 10^5^ cells/well. When 90–95% confluence was achieved, the cells were washed twice with uptake buffer at 37 °C. Then the cells were incubated with 200 µL coumarin-6-labeled PLGA NPs or LC-PLGA NPs in NaCl uptake buffer (140 mM NaCl, 25 mM Hepes/Tris, 5.4 mM KCl, 1.8 mM CaCl_2_, 0.8 mM MgSO_4_, and 5 mM glucose, pH 7.5), respectively. After 1 h incubation, the cells were washed three times with ice-cold uptake buffer. Then the cells were solubilized with 0.5 mL 1% (w/v) SDS/0.2 M NaOH, and the fluorescence intensity of coumarin 6 was determined with a fluorescence microplate reader with excitation/emission wavelengths set at 466 nm/504 nm. Protein was measured using the BCA protein assay kit (Thermo, USA).

As for the Na^+^-free buffer, the NaCl in the uptake buffer was iso-osmotically replaced with N-methyl-d-glucamine chloride; for Cl^–^-free buffer, NaCl, KCl, and CaCl_2_ were iso-osmotically replaced with sodium gluconate, potassium gluconate, and calcium gluconate, respectively. In addition, α-methyl-dl-tryptophan (α-MT) (2.5 mM) was used as a selective blocker of SLC6A14, and glycine (1 mM), a substrate of SLC6A14, was used as a competitive inhibitor to SLC6A14; L-carnitine (10 mM) was used as a competitive inhibitor to both SLC22A5 and SLC6A14.

### Colocalization studies

Cells were seeded on 12-mm coverslips in 24-well plates at a density of 0.5 × 10^5^ cells/well. When cells attached, the medium was replaced with NaCl uptake buffer containing coumarin 6 (5 µg/mL)-loaded nanoparticles for 15 min and 30 min, respectively. Cells were washed three times with ice-cold NaCl uptake buffer. The cells were fixed with 4% paraformaldehyde for 15 min at room temperature, and then permeabilized with PBS containing 0.1% Triton X-100 and 0.05% Tween 20 for 10 min at room temperature. After washing three times with PBS, the cells were incubated with primary antibodies at 4 °C overnight. After another three-time wash with PBST (PBS containing 0.05% tween 20), the cells were incubated with corresponding secondary antibodies for 1 h at room temperature. After another 3-time gentle wash with PBST, coverslips were placed sample-side down onto ProLong^®^ Diamond Antifade Mountant with DAPI (ThermoFisher, USA) on the glass slide. The slides were kept at room temperature for 12 h in the dark, and the cells were visualized under Nikon fluorescence microscope (Nikon, Japan).

### Assessment of the changes in transporter protein levels associated with LC-PLGA NPs uptake

MCF7 and Caco-2 cells were grown to 80–90% confluence in 10 cm dishes. After 0, 1, 2, 4, and 6 h treatment with blank 10%LC-PLGA NPs, cells were harvested and lysed in RIPA buffer, supplemented with 0.5 M EDTA and a protease inhibitor cocktail. The BCA assay kit was used to determine protein in samples. The protein extracts were analyzed by Western blot.

### Multi-table tournament (MTT) assay

The cell viability and proliferation assay was conducted using 3-(4, 5-dimethylthiazol-2-yl)-2, 5-diphenyltetrazolium bromide (MTT assay). Cells were seeded in 96-well plate at an appropriate density. After about 40% confluence was achieved, the cells were incubated with 5-FU, and 5-FU-loaded PLGA NPs and 5-FU-loaded LC-PLGA NPs at concentration range from 0.1 ng/mL to 20 µg/mL at 37 °C for 72 h in cell culture medium. After incubation, the medium was removed. MTT solution was added into wells and incubated at 37 °C for 4 h. The solution was then removed and DMSO was added to dissolve the contents of the wells, and the absorption was measured using microplate reader. IC_50_ was calculated using GraphPad Prism 6 software (GraphPad Software, Inc., La Jolla, CA).

### Spheroid cell culture

Spheroids were developed by non-adhesive liquid overlay method (Friedrich et al., 2009; Perche & Torchilin, 2012). HCT116 and HT29 cells, maintained in an incubator at 37 °C with 5% CO_2_ in 10-cm^2^ dishes until 70–80% confluency, were trypsinized, counted and suspended in media for spheroid culture. Ninety-six-well plate was coated with 50 µL of sterile 1.5% agarose solution in serum-free media, and was then cooled down to room temperature in 60 min to form a non-adhesive coating with a concave meniscus. Two-hundred microliters of PBS was added to each outer well to create an evaporation barrier. Hundred microliters of the cell suspension at a concentration of 3000 cells/well was seeded into each well and centrifuged at 1500 *g* for 15 min. The media in 96-well plate was replaced every two days with minimum spheroid disturbance.

#### LC-PLGA NPs penetration into tumor cell spheroid

After seeding, the spheroid was allowed to grow for four days. A pre-determined amount of coumarin-6-labeled LC-PLGA NPs was added to a final concentration of 5 µg/mL for 2 h. After that, the spheroids were collected and washed with excess of PBS to remove unassociated nanoparticles. They were transferred to slides with 200 µL of PBS and analyzed immediately on Nikon confocal microscope (Nikon, Tokyo, Japan) with a 10 × objective and 488 laser of Fluorescein isothiocyanate. Z-stack images were obtained at fixed intervals of 10 µm from periphery into the spheroid. Image J software was used to quantify the fluorescence intensity of coumarin 6.

#### Anti-tumor efficacy of LC-PLGA NPs in spheroid

HCT116 and HT29 spheroids were allowed to grow for 24 h. Spheroids were exposed to 1 µg/mL or 10 µg/mL 5-FU in either free form or in PLGA NPs or in 10%LC-PLGA NPs (PBS as control group). In all the cases, the spheroids were monitored for morphology and size by Nikon microscope and NIS-elements software 4.20 over the following 10 days.

### Statistical analysis

The data were presented as mean ± SD, and Student’s *t*-test was used to determine the statistical significance for the difference between the comparison groups.

## Results

### Preparation and characterisation of 5-FU-loaded LC-PLGA NPs

**Figure 1.**
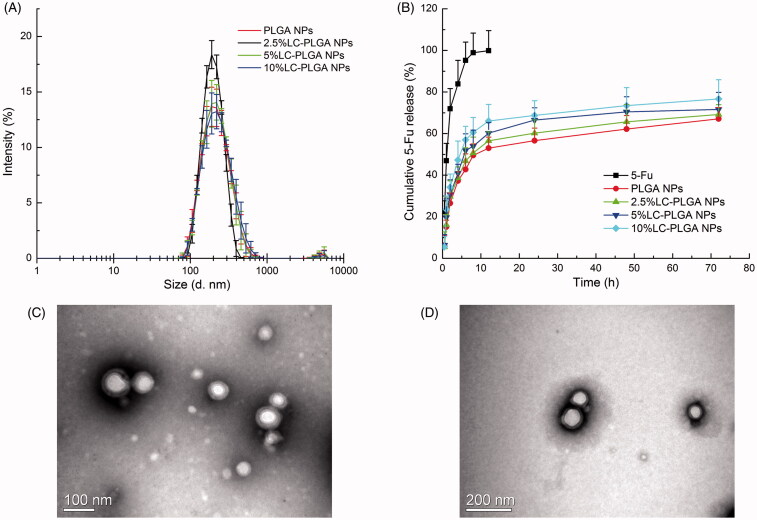
(A) Particle size and size distribution of PLGA NPs and LC-PLGA NPs, (*n* = 3); (B) *In vitro* release profiles of free 5-FU, 5-FU-loaded PLGA NPs and LC-PLGA NPs (*n* = 3); (C) TEM image of PLGA NPs; (D) TEM image of 10% LC-PLGA NPs.

LC-PLGA NPs were prepared using the nano-precipitation method (Li et al., 2017). As shown in Table S1 and Figure 1(A), the particle size of PLGA NPs, 2.5% LC-PLGA NPs, 5% LC-PLGA NPs and 10% LC-PLGA NPs was around 200 nm. Polydispersity index values of the particles were less than 0.2, indicating the particles were uniform. Increasing conjugation of l-carnitine from 0 to 10% in nanoparticles resulted in a slow gradual increase of zeta potential value (–2.16 ± 0.47, –1.42 ± 0.21, –1.08 ± 1.28, and –0.70 ± 0.38 mV, respectively). The entrapment of 5-FU in nanoparticles were more than 90%, and 5-FU-loading percentage was 4–5% in all nanoparticles. TEM images showed that both PLGA NPs (Figure 1(C)) and 10% LC-PLGA NPs (Figure 1(D)) were spherical and uniform. However, the particle size monitored by TEM was only about 100 nm, much smaller than by DLS (Table S1). The reason for discrepancy might be the different experimental conditions: for DLS, nanoparticles were dispersed in aqueous solution with an extensively stretched hydro-layer on the surface; in contrast, nanoparticles were dried prior to use for TEM, and the hydro-layer was collapsed and shrunken. In the *in vitro* release test (Figure 1(B)), compared to free 5-FU, 5-FU-loaded LC-PLGA NPs with various surface densities of l-carnitine conjugation (0, 2.5, 5, and 10%) exhibited a much slower but prolonged release of the drug. Coumarin 6 was used as a fluorescence marker to track the uptake process, and release profile of coumarin 6 from nanoparticles is shown in Figure S1, indicating a much lower and prolonged release compared to the coumarin 6 solution.

### Uptake features of LC-PLGA NPs in three cell lines

l-Carnitine is recognized as a substrate by the Na^+^-coupled transporter OCTN2 and the Na^+^/Cl^–^-coupled transporter ATB^0,+^ with different affinities. The involvement of both OCTN2 and ATB^0,+^ in the uptake of LC-PLGA NPs will be examined here.

**Figure 2.**
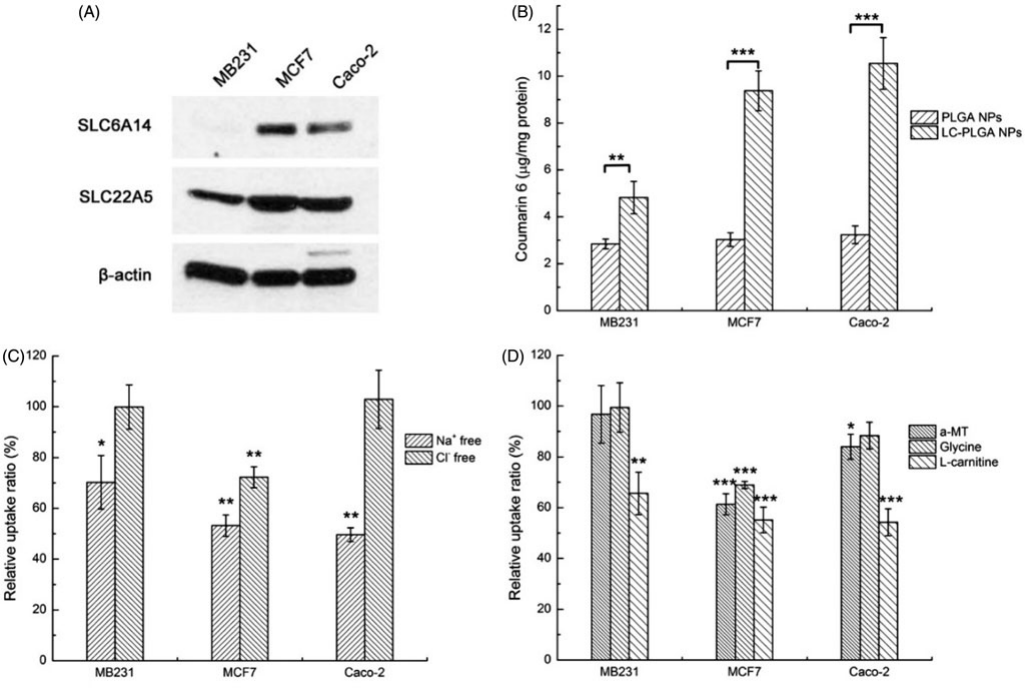
(A) Expression of OCTN2 (SLC22A5) and ATB^0,+^ (SLC6A14) proteins in MB231, MCF7 and Caco-2 cells, with β-actin as an internal control; (B) Uptake of coumarin 6 from bare nanoparticles (PLGA NPs) and L-carnitine conjugated nanoparticles (LC-PLGA NPs) in these three cell lines; (C) Effect of Na^+^ and Cl^–^ on the uptake of coumarin 6 from LC-PLGA NPs; (D) Effect of specific inhibitors (α-MT and glycine for ATB^0,+^, L-carnitine for OCTN2) on the uptake of coumarin 6 from LC-PLGA NPs. Data are shown as mean ± SD, *n* = 3. *, *p* < .05, **, *p* < .01, ***, *p* < .001, compared to uptake in NaCl buffer (C, D).

Three cell lines with different transporter expression patterns and different tissue origins were selected to test the possible involvement of OCTN2 and ATB^0,+^ in the uptake of LC-PLGA NPs: MB231 (OCTN2-positive and ATB^0,+^-negative, breast cancer cell line), MCF7 (both OCTN2- and ATB^0,+^-positive, breast cancer cell line), and Caco-2 (both OCTN2- and ATB^0,+^-positive, colon cancer cell line) (Figure 2(A)). In the uptake assay, conjugation of the nanoparticles with l-carnitine showed enhanced cellular uptake in all of the three cell lines compared to bare PLGA NPs (Figure 2(B)). It was clear that LC-PLGA NPs utilize OCTN2 as a target in MB231 cells for cellular uptake. In MCF7 and Caco-2 cells, the increased uptake could be attributed to both OCTN2 and ATB^0,+^.

The co-transporting ions in the uptake of nanoparticles targeting the transporters are critical (Luo et al., 2016; Kou et al., 2017). OCTN2 is a Na^+^-coupled transporter, and Na^+^ could act as a trigger for the OCTN2-mediated endocytosis in the uptake of LC-PLGA NPs. ATB^0,+^ is capable of mediating l-carnitine entry into cells in a Na^+^/Cl^–^-coupled manner, and therefore, both Na^+^ and Cl^–^ would be obligatory for ATB^0,+^-dependent uptake of nano-drug delivery vehicles. Here, Na^+^-free buffer and Cl^–^-free buffer were used to assess the involvement of both transporters. As shown in Figure 2(C), the uptake of LC-PLGA NPs in MB231 was significantly decreased in Na^+^-free buffer, confirming OCTN2-mediated uptake. As these cells did not express ATB^0,+^, removal of Cl^−^ in the uptake buffer did not have any effect on the uptake process. In MCF7 cells, both Na^+^-free buffer and Cl^–^-free buffer decreased the uptake of LC-PLGA NPs, indicating involvement of ATB^0,+^ in the uptake of LC-PLGA NPs. It is possible that OCTN2 was also involved in the uptake process as the function of this transporter is Na^+^-coupled, but it is difficult to make this conclusion unequivocally because the function of ATB^0,+^ is also dependent on Na^+^. In Caco-2 cells, the absence of Na^+^ decreased the absorption of LC-PLGA NPs, and the absence of Cl^−^ had no effect, suggesting the involvement of only OCTN2. Even though ATB^0,+^ is expressed in this cell line, it seems to make little or no contribution to the uptake process.

Because of the overlap in Na^+^-dependent between the two transporters, we used specific inhibitors of the transporters to delineate their involvement in the uptake of LC-PLGA NPs. α-Methyl-dl-tryptophan (α-MT) is a specific blocker for ATB^0,+^, and glycine is a substrate for ATB^0,+^. l-Carnitine is a substrate for both the transporters. As shown in Figure 2(D), α-MT and glycine showed on effect on the uptake of LC-PLGA NPs in MB231. These findings agreed with the data that these cells did not express ATB^0,+^. However, free l-carnitine decreased the uptake, confirming the participation of OCTN2. In MCF7 cells, all the three inhibitors decreased the uptake of LC-PLGA NPs, indicating the participation of both transporters in the uptake process.

Caco-2 cells expressed both OCTN2 and ATB^0,+^. Therefore, we used glycine as a competitive substrate for the transporter to determine the participation of the transporter in the uptake process. We found no effect of glycine on the uptake of LC-PLGA NPs in Caco-2 cells with 1 h incubation (Figure S2). However, when the incubation time was extended to 3 h, glycine decreased the uptake of LC-PLGA NPs, supporting a role of ATB^0,+^ in the uptake of LC-PLGA NPs. It is likely that OCTN2 is the major contributor to the uptake with the contribution but ATB^0,+^ being relatively minor.

### Colocalisation of LC-PLGA NPs and OCTN2/ATB^0,+^

**Figure 3:**
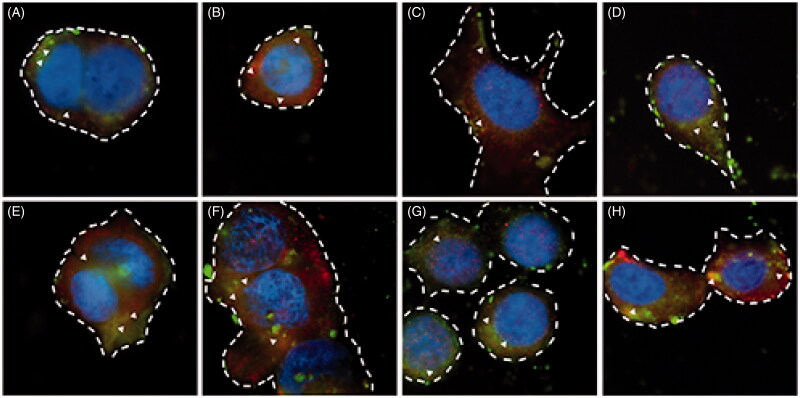
, Fluorescent images of the colocalisation for LC-PLGA NPs and OCTN2/ATB^0,+^ in MCF7 cells (A, B, E, F) and Caco-2 cells (C, D, G, H). Coumarin 6 (green) was used to label LC-PLGA NPs, and Alexa Fluor^®^ 594 (red) conjugated secondary antibody was used to mark OCTN2 or ATB^0,+^, and DAPI (blue) was used to show the nucleus. A, B, C, D for 15 min incubation, and E, F, G, H for 30 min incubation. In MCF7 cells, A and E show the colocalisation of LC-PLGA NPs with ATB^0,+^, and B and F show the colocalisation of LC-PLGA NPs with OCTN2; In Caco-2 cells, C and G show the colocalisation of LC-PLGA NPs with ATB^0,+^, and D and H show the colocalisation of LC-PLGA NPs with OCTN2. Arrowheads show the colocalisation of LC-PLGA NPs and transporters (yellow, green + red). Dashed lines mark the cell borders.

To further characterize the OCTN2- and ATB^0,+^-mediated absorption process of LC-PLGA NPs, we performed colocalization studies for the LC-PLGA NPs and the transporters. As shown in Figure 3, coumarin 6 (green) was used to label LC-PLGA NPs, and Alexa Fluor^®^ 594-(red) conjugated secondary antibody was used to identify OCTN2 or ATB^0,+^. DAPI (blue) was used to mark the nucleus. After 15 min incubation at 37 °C (Figure 3(A–D)), both in MCF7 cells (Figure 3(A, B)) and Caco-2 cells (Figure 3(C, D)) cells, there were yellow (red + green, arrowheads) signals for LC-PLGA NPs and ATB^0,+^ (Figure 3(A, C)) and also for LC-PLGA NPs and OCTN2 (Figure 3(B, D)). These data confirmed that both ATB^0,+^ and OCTN2 were involved in the uptake of LC-PLGA NPs in both cells. The signal was much stronger after 30 min incubation (Figure 3(E–H): 3(E) and 3(F) for MCF7; 3 G and 3H for Caco-2; 3E and 3 G for ATB^0,+^; 3 F and 3H for OCTN2). Combined with the above results, we concluded that LC-PLGA NPs target both ATB^0,+^ and OCTN2. It was interesting that ATB^0,+^ was also involved in the uptake of LC-PLGA NPs even though the affinity of this transporter for l-carnitine is almost 2 orders of magnitude less than that known for OCTN2. The multivalent interaction between LC-PLGA NPs and the two transporters is likely to enhance the cellular uptake of the nanoparticles if both transporters are expressed in the same cells (Figure S5). A firm binding could be achieved by LC-PLGA NPs through multivalent anchoring to facilitate nanoparticle entry into cells.

### Impact of LC-PLGA NPs on cellular protein levels for the transporters

Transporters formed a complex with targeting nanoparticles and then got endocytosed into the cells. Except for caveolin-mediated endocytosis where the transporters get recycled back into the plasma membrane, in other types of endocytic processes, the complex fuses with lysosomes where the transporters get degraded, thus reducing the cellular levels of the transporter protein (Kou et al., 2013). Here, we studied the cellular protein levels for OCTN2 and ATB^0,+^ in both MCF7 cells and in Caco-2 cells following incubation with LC-PLGA NPs. As shown in Figures S3 and S4, the amount of the transporter protein decreased in a time-dependent manner in both cell lines, confirming the intracellular degradation of OCTN2 and ATB^0,+^ following the endocytosis process.

### Characterization of colon cells

**Figure 4.**
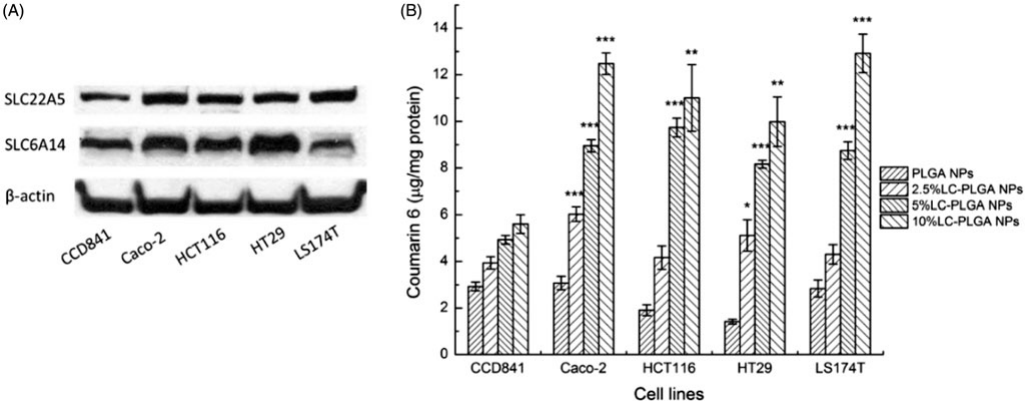
(A) Expression of OCTN2 (SLC22A5) and ATB^0,+^ (SLC6A14) in the normal colon cell line (CCD841) and colon cancer cell lines (Caco-2, HCT116, HT29, LS174T); (B) Uptake of coumarin 6 from LC-PLGA NPs with different ligand density (0 to 10%) in colon cells. Data are shown as mean ± SD, *n* = 3. *, *p* < .05, **, *p* < .01, ***, *p* < .001, compared to uptake in normal colon cells (CCD841).

The data from the present studies show that LC-PLGA NPs target both OCTN2 and ATB^0,+^ for enhanced cellular uptake. This feature can be exploited as a strategy to deliver therapeutic drugs in to selective cells that express high levels of both transporters. To assess the validity of this strategy for colon cancer therapy, we examined the expression of both transporters in one normal colon epithelial cell line (CCD841) and four colon cancer cell lines (Caco-2, HCT116, HT29, and LS174T) using western blot (Figure 4(A)). Both transporters were found at much higher levels in cancer cells than in the normal cell line. These data indicated that LC-PLGA NPs could be used to target OCTN2 and ATB^0,+^ in colon cancer cells for improved delivery of chemotherapeutic agents.

### Uptake assay of LC-PLGA NPs with different ligand density in colon cells

In our previous study, we found that the surface density of l-carnitine in LC-PLGA NPs had significant impact on the cellular uptake of nanoparticles in the intestinal tract (Kou et al., 2017). Here we examined whether a similar effect occurs even for the uptake of LC-PLGA NPs in colon cells. As shown in Figure 4(B), with the ligand density increasing from 0 to 10%, the uptake in all the cells increased. As the expression of OCTN2 and ATB^0,+^ was higher in cancer cells than in normal cells, the uptake for LC-PLGA NPs was also higher in cancer cells than in normal cells. With the ligand density from 0 to 10%, the uptake of LC-PLGA NPs in normal cell increased less than 2-fold, but the magnitude of the effect was much higher in cancer cells.

### MTT assay

**Figure 5.**
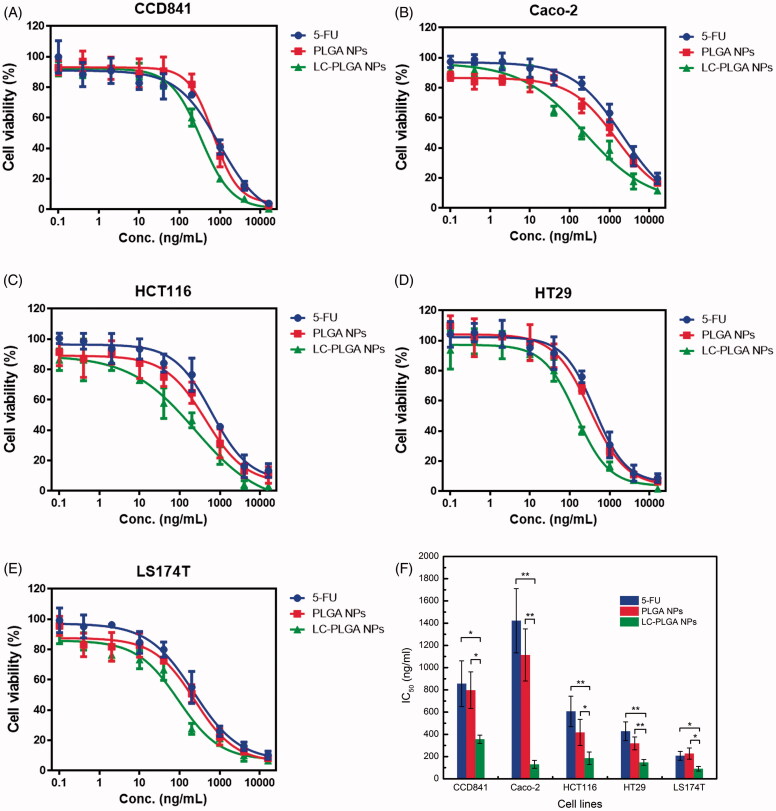
The MTT assay for 5-FU, 5-FU-loaded PLGA NPs and 5-FU-loaded LC-PLGA NPs in CCD841 (A); Caco-2 (B); HCT116 (C); HT29 (D); and LS174T (E); (F), the calculated IC50 values. Data are shown as mean ± SD, *n* = 3. *, *p* < .05, **, *p* < .01.

5-FU, a drug widely used for colon cancer therapy, was taken here as a model drug for MTT assay to assess the potential utility of LC-PLGA NPs for drug delivery in colon cancer cells. *In vitro* cytotoxicity experiments of free 5-FU, 5-FU-loaded PLGA NPs and 5-FU-loaded 10%LC-PLGA NPs were performed in one normal cancer cell line and four colon cancer cell lines; the dose-response curves are presented in Figure 5. In CCD841 cells, 5-FU-loaded PLGA NPs showed less cytotoxicity compared to free 5-FU, but 5-FU-loaded LC-PLGA NPs showed increased cytotoxicity. In the other four cancer cells, nanoparticles always had higher cytotoxicity than free drug, but LC-PLGA NPs had the greatest cytotoxicity effect.

### LC-PLGA NPs penetration of cancer cell spheroids

**Figure 6.**
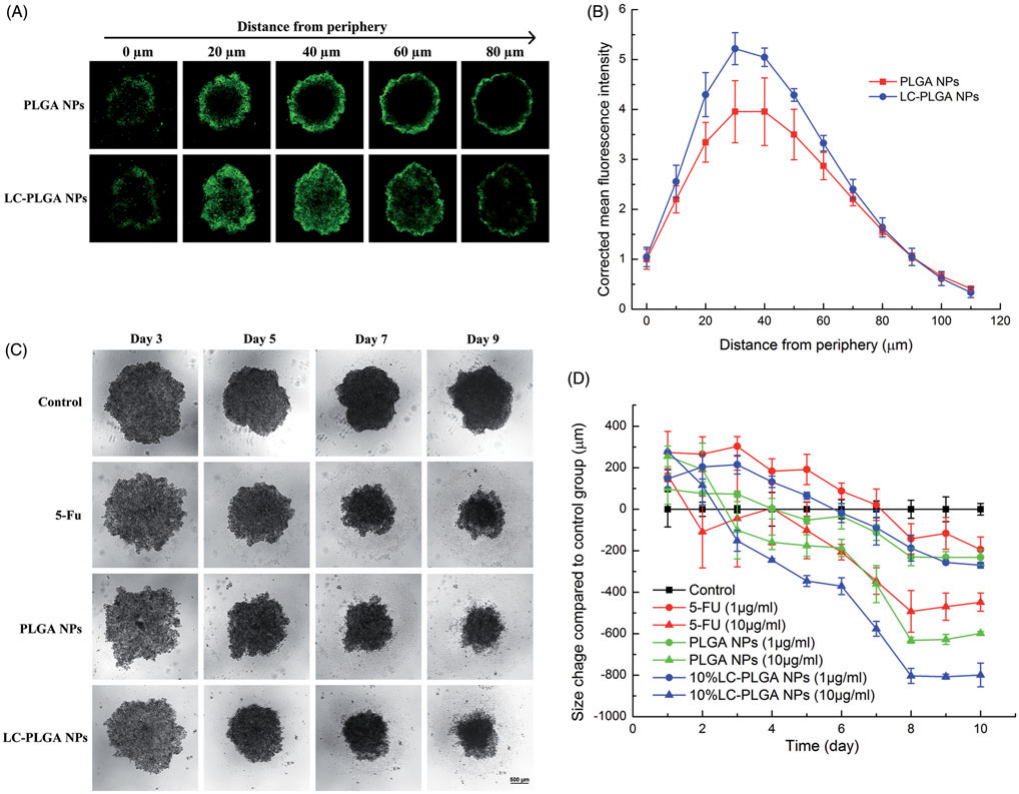
(A) Nanoparticle penetration of spheroids. Z-stack images taken by confocal microscopy showing penetration of coumarin 6-labeled PLGA NPs and LC-PLGA NPs in HCT116 spheroids. Green color indicates coumarin 6-labeled nanoparticles; (B) Corrected coumarin 6 flourescence intensity represents the nanoparticles in spheroids from periphery to the inner layer (*n* = 3); (C) Morphological change in HCT116 spheroids during 10-day treatment with 10 µg/mL of free 5-FU, 5-FU-loaded PLGA NPs and 5-FU-loaded LC-PLGA NPs; (D) Compared to control group, the size change of HCT116 spheroids treatment with 1 µg/mL and 10 µg/mL of free 5-FU, 5-FU-loaded PLGA NPs and 5-FU-loaded LC-PLGA NPs (*n* = 3).

Cancer cell spheroids have been proposed as models for the evaluation of nano-drug delivery system (Breslin & O’Driscoll, 2013; Wu et al., 2017). The penetration of nanoparticles into spheroids is a critical step for drug delivery efficiency. Here we used HCT116 and HT29 cell spheroids to evaluate the superiority of LC-PLGA NPs in drug delivery into solid tumors. The tumor spheroids were incubated with coumarin-6-labeled PLGA NPs and LC-PLGA NPs for 2 h, and the nanoparticle penetration and distribution in spheroids were observed with laser scanning confocal microscopy. The confocal images from the periphery to the core of the spheroids and the corrected coumarin 6 fluorescence intensity vs. distance are shown in Figure 6(A, B) (HCT116 spheroid) and Figure S6 (HT29 Spheroid). There was no obvious difference in penetration ability between PLGA NPs and LC-PLGA NPs in both HCT116 and HT29 spheroids. The peak presence of PLGA NPs and LC-PLGA NPs occurred at 30 µm in both HCT116 and HT29 spheroids, and the fluorescence decreased with distance towards the core. However, quantitative measurement of fluorescence showed a greater uptake with LC-PLGA NPs than PLGA NPs in both the cases, indicating that conjugated l-carnitine increased the cellular uptake of nanoparticles in solid spheroid models.

### Anti-tumor efficiency in 3D spheroids

Unlike classical cell culture-based models, spheroids prominently mirror the 3D cellular context and therapeutically relevant pathophysiological gradients of *in vivo* tumors. Accordingly, the 3D spheroids model is increasingly recognised as a suitable tool to evaluate the efficacy of nano-drug carriers for drug delivery (Breslin & O’Driscoll, 2013; Wu et al., 2017). Therefore, we used HCT116 and HT29 spheroids to evaluate the *in vitro* anti-tumor efficiency of LC-PLGA NPs as a delivery system for 5-FU. With two doses of 5-FU (1 µg/mL and 10 µg/mL), we compared the efficacy of the drug in the free form and when encapsulated in bare PLGA NPs or in LC-PLGA NPs. The morphology of spheroids and size change compared to the control group are shown in Figure 6(C, D) (HCT116) and Figure S7 (HT29). After treatment with the drug, the spheroids were disassembled and decreased in size from periphery to core, but the phenomenon did not occur in the absence of the drug. With 10 µg/mL of 5-FU, nanoparticles showed significantly greater anti-tumor efficacy than the free drug, and LC-PLGA NPs showed even greater anti-tumor efficacy.

## Discussion

Here we have described a novel feature of l-carnitine conjugated nanoparticles (LC-PLGA NPs) in tumor cell-targeted drug delivery. Nanoparticles themselves are taken up by mammalian cells by nonselective endocytosis, but chemical modification of the surface of these nanoparticles in such a manner that these nanoparticles are recognized by tumor cell-specific cell-surface proteins would potentially enhance the efficiency of the endocytic process and also tumor cell selectivity. l-Carnitine is a transportable substrate for two plasma membrane transporters: OCTN2, a Na^+^-coupled transporter that recognizes l-carnitine with high affinity (K_m_ ∼10 μM), and ATB^0,+^, a Na^+^- and Cl^–^-coupled transporter that recognizes l-carnitine with relatively low affinity (K_m_ ∼ 800 μM). In the present study, we have shown that anchoring l-carnitine to the surface targets the nanoparticles to both transporters. We have already shown that LC-PLGA NPs are able to increase oral delivery of therapeutic agents by enhancing the interaction of the NPs with OCTN2 expressed on the lumen-facing membrane of the intestinal enterocytes (Kou et al., 2017). Here we assessed the potential of LC-PLGA NPs for drug delivery into cancer cells. The rationale for these studies is the fact that cancer cells express high levels of OCTN2 and also ATB^0,+^, both of which are known to recognize l-carnitine as substrates.

The data have shown convincingly that linking l-carnitine to the surface of the nanoparticles facilitates drug delivery into tumor cells by targeting both OCTN2 and ATB^0,+^. Demonstration of the dual targeting mechanism was feasible because of the availability of appropriate cancer cell lines that express OCTN2 and ATB^0,+^, either singly or together. The human breast cancer cell lines MCF7 and MB231 show differential expression of ATB^0,+^, the former being positive for robust expression of ATB^0,+^ and the latter being completely negative for ATB^0,+^ expression. However, both cell lines express OCTN2. Caco-2 is a human colon cancer cell line that is positive for both OCTN2 and ATB^0,+^. In all these cell lines, uptake of encapsulated drugs in LC-PLGA NPs was dependent on Na^+^; as both OCTN2 and ATB^0,+^ are both Na^+^-coupled, the dependence of uptake on Na^+^ does not differentiate the involvement between OCTN2 and ATB^0,+^. But we were able to differentiate between these two transporters by other means. First, we monitored the effect of Cl^–^ on the uptake process. This anion is obligatory only for ATB^0,+^ but not for OCTN2. As such, removal of Cl^–^ from the uptake medium decreased the uptake of drugs from LC-PLGA NPs in ATB^0,+^-positive cell lines (MCF7 and Caco-2) but not in ATB^0,+^-negative cell line (MB231). Second, we used transporter-specific substrates that can compete with l-carnitine on the surface of LC-PLGA NPs for interaction with the two transporters. l-Carnitine is a substrate for both OCTN2 and ATB^0,+^, but glycine is a substrate only for ATB^0,+^. As such, excess amount of l-carnitine in the uptake medium decreased the uptake of drugs encapsulated in LC-PLGA NPs in all cell lines but excess glycine in the uptake medium decreased the uptake only in the cell lines that are positive for ATB^0,+^. Third, we also used a selective blocker of ATB^0,+^, namely α-MT; this blocker behaved similar to glycine by interfering with drug uptake from LC-PLGA NPs selectively in ATB^0,+^-positive cell lines.

There are two aspects of drug uptake from LC-PLGA NPs that need to be emphasized. First is the relevance of the relative affinities of OCTN2 and ATB^0,+^ for l-carnitine. The affinity of OCTN2 for L-carnitine is almost two orders of magnitude greater than that of ATB^0,+^; yet, LC-PLGA NPs are targeted to both transporters. This may not necessarily mean that the affinity of a given transporter for the cell-surface ligand on the NPs has no relevance; as the involvement of the two transporters in the present study was assessed by drug uptake rather than by binding of NPs to the surface of the cells, the technique monitors the overall efficiency of the NP entry into cells that includes binding and endocytosis. The driving forces for OCTN2 and ATB^0,+^ are also different; OCTN2 is driven by an electrochemical Na^+^ gradient whereas ATB^0,+^ is driven by an electrochemical Na^+^ gradient and also by the Cl^–^ gradient. It is true that these driving forces are not only for the transmembrane transfer of the substrates but also for the transporter-mediated endocytosis. The second significant feature is the potential role of polyvalent interaction of the cell-surface ligand of the nanoparticles with the transporters. The uptake of encapsulated drugs from LC-PLGA NPs increased as the surface density of l-carnitine on these NPs increased. These findings lead to the speculation that the presence of multiple copies of the ligand on the surface of the nanoparticles might recruit multiple copies of the transporters to bind one molecule of the nanoparticle, a phenomenon that might likely enhance the transporter-assisted endocytosis (Figure S5).

The novelty of the studies presented here is obvious. It is easy to understand the logic behind the modification of the surface of the NPs to target them to specific proteins on the surface of the target cells (Li et al., 2016; Luo et al., 2016; Luo et al., 2017; Shao et al., 2014; Hayashi et al., 2016). But here conjugation of l-carnitine to the surface of the NPs leads to dual targeting. This means that judicial selection of the ligand for conjugation could be used to maximise the cellular uptake of the nanoparticles. It has been recognized that conversion of monovalent interaction to multivalent interaction can prominently enhance the internalization (Muro, 2012). The force of monovalent binding is expected to be weak. During multivalent binding, the interaction of NPs with the surface of the target cells is stronger to enhance endocytosis (Figure S5). In the present study, LC-PLGA NPs exhibit dual targeting, binding to both OCTN2 and ATB^0,+^; this probably provide more opportunities for the formation of multivalent binding between LC-PLGA NPs and cell surface.

Colocalization experiments have clearly shown that the LC-PLGA NPs bind to OCTN2 and ATB^0,+^. Interestingly, the transporter protein levels decrease in a time-dependent manner when the cells are exposed to these NPs. These findings suggest that the transporter protein is not recycled following endocytosis, but instead gets degraded. The endocytic mechanisms of LC-PLGA NPs have been investigated in our previous study (Kou et al., 2017), suggesting that clathrin-mediated, caveolin-mediated endocytosis, and macropinocytosis were involved in the endocytic process. Except for caveolin-mediated endocytosis, the endosomes containing nanoparticles and transporters would get fused with lysosomes for clathrin-mediated endocytosis and macropinocytosis, resulting in drug release and transporter degradation (Kou et al., 2013). However, new proteins would be translated and transferred to cytomembrane in the next few hours, and thus the physiological properties of cells would not be affected. When protein level diminished, the side effect on normal cells would be still less because the new proteins will be reproduced in the next few hours, and LC-PLGA NPs would still prefer to bind to both OCTN2 and ATB^0,+^ overexpressed colon cancer cells, rather than normal cells. The similar phenomenon was also found in other studies, and the temporary decrease of target proteins did not affect the following tumor targeting and make unexpected side effect (Li et al., 2017; Luo et al., 2016).

Compared to normal tissues, tumors always have an increased demand for nutrition, like glucose and amino acids, to support their rapid growth and proliferation (Ganapathy et al., 2015). To match that, several plasma membrane transporters, obligated to transport these nutrition, such as GLUT1, LAT1, and ATB^0,+^, were evidenced to be overexpressed in tumor site, providing ideal targets for tumor-selective drug delivery (Ganapathy et al., 2015). One of the potential applications of drug delivery nanosystems in cancer therapy to enhance tumor cell-specific exposure to cytotoxic drugs with relatively little exposure to normal cells. This goal could be achieved by selecting the cell-surface molecular targets for the ligand-conjugated NPs. If such targets are expressed on tumor cells at much more density than in normal cells, it would maximise tumor cell selectivity for drug delivery. This happens to be the case with the molecular targets employed in the present study. Colon cancer is one of the most common cancers worldwide. It has been reported that ATB^0,+^ is expressed at low levels in normal colon, but its expression is upregulated in colon cancer (Gupta et al., 2005). Present studies corroborate these published reports. There is no consensus from published reports with regard to changes in the expression of OCTN2 in cancer (Calcagno et al., 2006; Fujiya et al., 2011; Martini et al., 2012; Scalise et al., 2012). However, our studies show increased expression of the transporter in colon cancer cells than in normal cells. Thus, LC-PLGA NPs could be potentially useful for the delivery of chemotherapeutic drugs for colon cancer.

*In vitro* 3D cell spheroid models mimic the native tumor environment, mirroring the situation observed in patients or in preclinical tumor-bearing animals. A key feature of this model is its utility to investigate the complexity of treatment for a solid tumor. Here we used 3D cell spheroids for the colon cancer cell lines HCT116 and HT29 to compare the efficacy of LC-PLGA NPs vs. PLGA NPs on tumor uptake of cytotoxic drugs and consequent anti-tumor efficiency. Compared to unmodified PLGA NPs, LC-PLGA NPs showed enhanced drug delivery in spheroids derived from both the cell lines (Figures 6(A, B) and S6). Although there were no significant differences in penetration between LC-PLGA NPs and bare PLGA NPs in spheroids, the increased uptake of LC-PLGA NPs contributed to the higher anti-tumor efficiency (Figures 6(C, D) and S7).

OCTN2 is expressed throughout the small intestine while ATB^0,+^ is expressed preferentially in the ileum and colon. Therefore, one of the potential pitfalls in the use of LC-PLGA NPs for drug delivery to treat colon cancer is the likely interaction of the drug-loaded NPs with OCTN2 in the small intestine prior to reaching the targeted site, namely the colon. However, this pitfall can be addressed by appropriate changes in the design of the nanoparticles. LC-PLGA NPs could be packaged into a commercial enteric capsule or coated with specific enteric polymer for colon delivery (Wang et al., 2015; Tummala et al., 2016). The formulation of these NPs is such that the LC-PLGA NPs do not get access to OCTN2 expressed in the small intestine, however, they get released in the colonic lumen where they are free to interact with OCTN2 and ATB^0,+^ expressed in the colon.

In recent years, several kinds of nano-drug delivery systems conjugated with diverse ligands, such as antibodies, peptides, and other small molecules, have been examined for colon cancer therapy with enhanced specificity. One recent study has reported identification of four biomarkers in colorectal cancer tissue from 280 patients: carcinoembryonic antigen expressed in colorectal cancer 98.8% more than in normal tissue, tumor-associated glycoprotein-72 at 79%, folate receptor-α at 37.1%, and EGFR at 32.8% (Tiernan et al., 2013). These cell-surface proteins offer potential molecular targets for nano-drug delivery to colon cancer. Cortez et al. (2007) described a polymer capsule conjugated with the humanised A33 mAb (huA33 mAb) formed by a layer-by-layer method, which has shown great promise in the treatment of colon cancer. However, the major limitation of antibodies is the large size, stability, and complexity. Small peptides offer an alternative due to their small size and ease of attachment to nanoparticles. HPMA-copolymer conjugated with oligopeptide G11 has shown high cellular uptake *in vitro* in colon cancer cells with EGFR (epidermal growth factor receptor) overexpression (Kopansky et al., 2011). Another example of the use of peptide ligands for nano-drug delivery is the tumor necrosis factor-related apoptosis-inducing ligand and the peptide RPMrel (CPIEDRPMC) (Cisterna et al., 2016). Small molecules are much more stable than biomacromolecules and easy to be conjugated to nanoparticles. Like the L-carnitine we used here, the certain structural features of carnitine are ideal for its conjugation onto a surface of nanoparticles. LC-PLGA NPs selectively target to OCTN2 and ATB^0,+^ overexpressed in colon cancer cells. The present studies also add OCTN2 and ATB^0,+^ to this list as potential molecules on colon cancer cells to target nanoparticles with modification of their surface with L-carnitine as the ligand for both transporters.

## Conclusions

Conjugation of PLGA nanoparticles with L-carnitine on the surface targets them to two plasma membrane transporters, OCTN2 and ATB^0,+^, both of which are expressed at higher levels in colon cancer cells than in normal cells. This selective targeting enhances the exposure of colon cancer cells to cytotoxic drugs administered encapsulated in LC-PLGA NPs. The delivery of the encapsulated drugs into cancer cells involves transporter-mediated endocytosis. These findings demonstrate the feasibility of using selective ligands on the surface of the nanoparticles to target them to selective cell-surface proteins expressed highly in cancer cells for tumor cell-selective drug delivery.

## Supplementary Material

IDRD_Sun_et_al_Supplemental_Content.docx
