# Supplementary material for: Dual targeting of l-carnitine-conjugated nanoparticles to OCTN2 and ATB0,+ to deliver chemotherapeutic agents for colon cancer therapy
Source: Drug Deliv. 2017 Sep 15;24(1):1338–49. doi: 10.1080/10717544.2017.1377316 (PMC8241000; doi:10.1080/10717544.2017.1377316)
Supplement: IDRD_Sun_et_al_Supplemental_Content.docx [file IDRD_A_1377316_SM0584.docx]

Supporting Information

Dual Targeting of L-carnitine-conjugated Nanoparticles to OCTN2 and ATB^0,+^ to Deliver Chemotherapeutic Agents for Colon Cancer Therapy

Longfa Kou^1,2^, Qing Yao^1^, Sathish Sivaprakasam^2^, Qiuhua Luo^1^, Yinghua Sun^1^, Qiang Fu^1^, Zhonggui He^3^, Jin Sun^1,^ *, and Vadivel Ganapathy^2,^ *

^1^Municipal Key Laboratory of Biopharmaceutics, Wuya College of Innovation, Shenyang Pharmaceutical University, Shenyang, China

^2^Department of Cell Biology and Biochemistry, Texas Tech University Health Sciences Center, Lubbock, TX, USA

^3^Department of Pharmaceutics, Wuya College of Innovation, Shenyang Pharmaceutical University, Shenyang, China

*Corresponding authors:

Prof. Vadivel Ganapathy, Department of Cell Biology and Biochemistry, Texas Tech University Health Sciences Center, 3610 4th Street, Lubbock, TX, 79430, USA. Tel: +1-(806)743-2518; Fax: +1-(806)743-2990; Email: [vadivel.ganapathy@ttuhsc.edu](mailto:vadivel.ganapathy@ttuhsc.edu)

Prof. Jin Sun, Municipal Key Laboratory of Biopharmaceutics, Wuya College of Innovation, Shenyang Pharmaceutical University, No. 103 Wenhua Road, Shenyang, 110016, China. Tel: +86-24-23986325; Fax: +86-24-23986320; Email: [sunjinsypharm@163.com](mailto:sunjinsypharm@163.com)

Supporting Information

Dual Targeting of L-carnitine-conjugated Nanoparticles to OCTN2 and ATB^0,+^ to Deliver Chemotherapeutic Agents for Colon Cancer Therapy

# Materials and methods

## Preparation of L-carnitine-conjugated nanoparticles

Briefly, PLGA (10 mg), stearoyl-L-carnitine (1 mg), and 5-fluorouracil (0.5 mg) were dissolved in acetone (2 mL). The solution of drug and copolymer was added drop-wised into distilled water with 1% PVA (10 mL) with constant stirring. The solvent was then removed via overnight evaporation at room temperature. The resulting nanoparticle solution was filtered through 0.45 µm filter, and then centrifuged at 148,000 rpm for 30 min to collect nanoparticles. The supernatant was discarded and nanoparticles were resuspended in distilled water. After washing three times, the sediment was freeze-dried. Nanoparticles with varying amounts of carnitine conjugation were prepared by varying the ratio stearoyl-L-carnitine/PLGA. Blank nanoparticles or non-modified (PLGA NPs) nanoparticles were both prepared using the same procedure in the absence of 5-fluorouracil or stearoyl-L-carnitine, respectively. Coumarin-6-loaded nanoparticles were prepared in a similar manner by adding coumarin-6 to acetone instead of 5-fluorouracil. Coumarin 6 was used as fluorescent marker to monitor the efficiency of nanoparticles in drug delivery, and 5-fluorouracil was used as model drug to treat colon cancer.

***Characterization of L-carnitine conjugated nanoparticles***

*Size and zeta potential*

The average particle size and polydispersity index (PDI) were measured by dynamic light scattering (DLS) (NanoZetasizer, Malvern Co., UK), and the zeta potential of nanoparticles was determined using NanoZetasizer (NanoZetasizer, Malvern Co., UK).

*Particle morphology*

The shape and morphology of L-carnitine conjugated PLGA nanoparticles was examined using transmission electron microscopy (TEM) (Hitachi H-600, Tokyo, Japan). A drop of suitably diluted nanoparticle solution was deposited onto a carbon-coated copper grid to form a thin film. After the film was dried, it was counterstained with 1% phosphotungstic acid. Any excess solution was removed with a filter paper. The sample was dried at room temperature and then examined using TEM.

*Drug-loading (DL) and entrapment efficiency (EE)*

A protein concentrator (3K MWCO, Thermo Fisher Scientific Inc.) was used to separate the free drug from the nanoparticle solution. Briefly, the nanoparticle solution (1 mL) was placed into the concentrator sample chamber and then centrifuged for 10 min at 3000 rpm. This procedure concentrated the drug-loaded nanoparticles in the chamber, and the unentrapped drug was in the filtrate. The fraction of entrapment of the drug was determined by estimated the untrapped drug in the filtrate and comparing it with the total amount of drug added originally for the entrapment. The drug 5-FU in the filtrate was measured spectrophotometrically (265 nm). Acetone was used to lyse the nanoparticles and release the loaded 5-FU, which was then measured to confirm the total amount of the drug added originally.

The DL and EE of 5-FU in NPs were calculated as follows:

DL (%) = (amount of 5-FU in NPs/amount of 5-FU-containing NPs) × 100%.

EE (%) = (1-amount of 5-FU untrapped/amount of total 5-FU added into formulation) × 100%.

***In vitro release***

The in vitro release of 5-fluorouracil from nanoparticles was assessed using a dialysis method. In brief, 2 mL nanoparticle solution was placed into a snakeskin dialysis tubing (10K MWCO, 35 mm, Thermo Fisher Scientific Inc.) and incubated in release medium (30 mL, PBS, pH 7.4) at 37 °C with constant shaking (orbital shaker, 100 rpm). At designated intervals, 0.5 mL samples were taken for analysis and replaced with the same volume of fresh medium. The 5-FU content was determined by UV spectrophotometrically.

The in vitro release of coumarin 6 from nanoparticles was conducted using a similar method, just replacing the pH 7.4 PBS with pH 7.4 PBS containing 2% cremophor EL to increase the solubility of coumarin 6 in release medium. The coumarin 6 solution was prepared using PBS containing 10% cremophor EL and 10% ethanol. The coumarin 6 contend was determined with a fluorescence microplate reader with excitation/emission wavelengths set at 466 nm/504 nm.

Table S1. Physicochemical characterization of LC-PLGA NPs.

|  | Size (nm) | PDI | Zeta potential (mV) | EE (%) | DL (%) |
| --- | --- | --- | --- | --- | --- |
| PLGA NPs | 203.5 ± 1.3 | 0.167 ± 0.015 | -2.16 ± 0.47 | 93.89 ± 2.46 | 4.47 ± 0.12 |
| 2.5%LC-PLGA NPs | 188.6 ±3.0 | 0.064 ± 0.014 | -1.42 ± 0.21 | 92.47 ± 3.52 | 4.40 ± 0.17 |
| 5%LC-PLGA NPs | 207.3 ± 1.6 | 0.166 ± 0.017 | -1.08 ± 1.28 | 92.86 ± 2.81 | 4.42 ± 0.13 |
| 10%LC-PLGA NPs | 209.1 ± 2.6 | 0.158 ± 0.009 | -0.70 ± 0.38 | 91.22 ± 2.87 | 4.34 ± 0.14 |


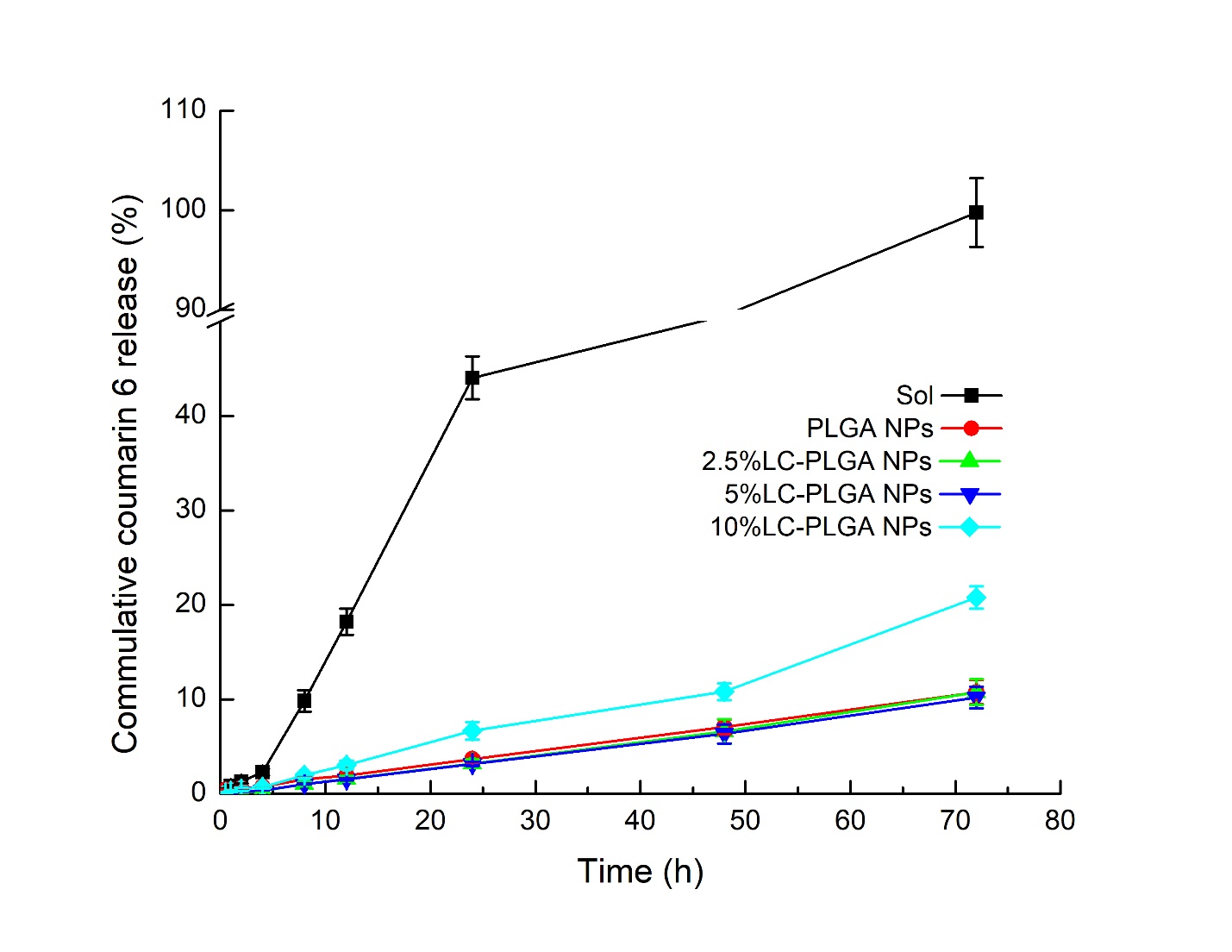


Figure S1. In vitro release profiles of coumarin 6 solution, coumarin 6-loaded PLGA NPs and LC-PLGA NPs (n = 3).


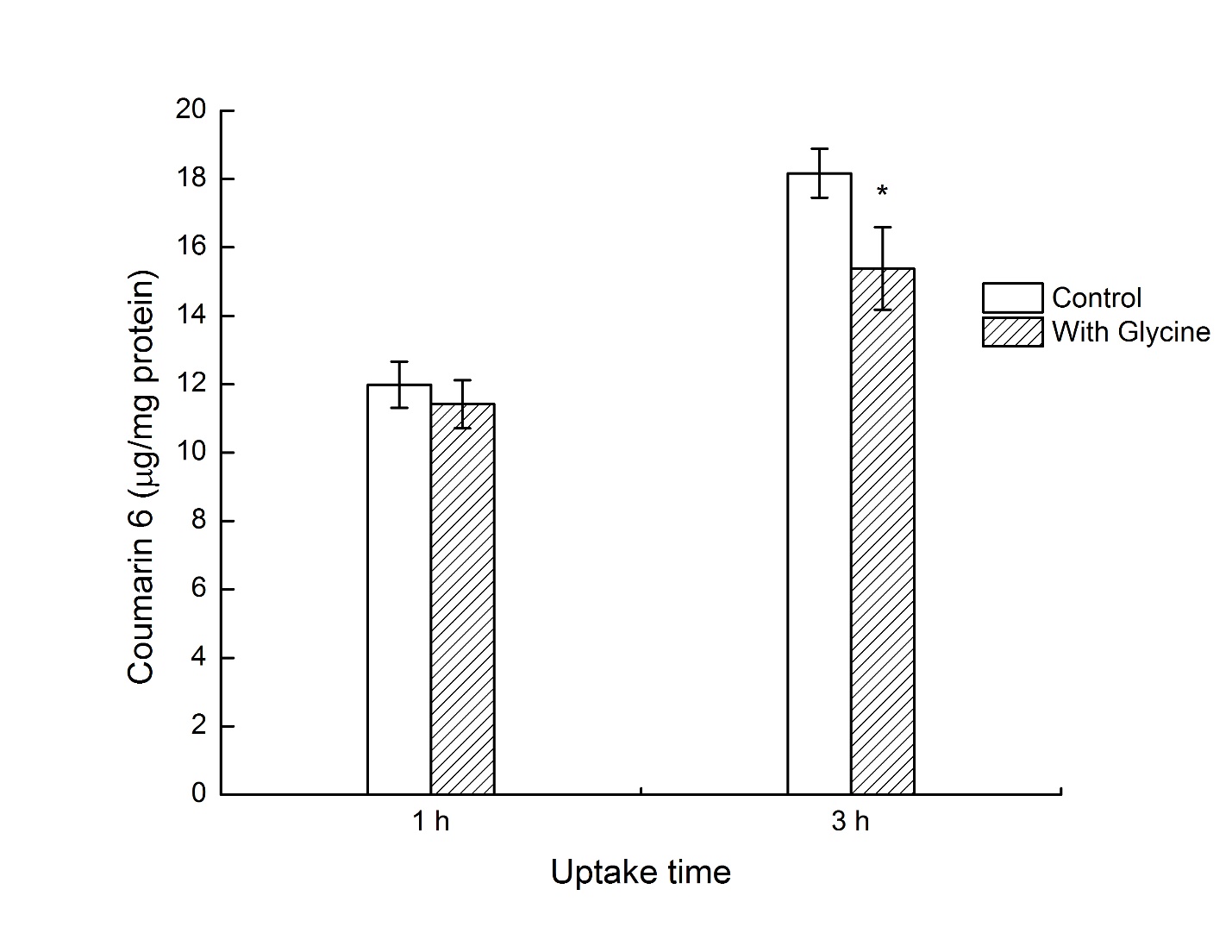


**Figure S2**. Uptake of coumarin 6 from LC-PLGA NPs in Caco-2 cells with two different periods of exposure. Data are shown as mean ± SD, n = 3. *, P < 0.05, vs the control.


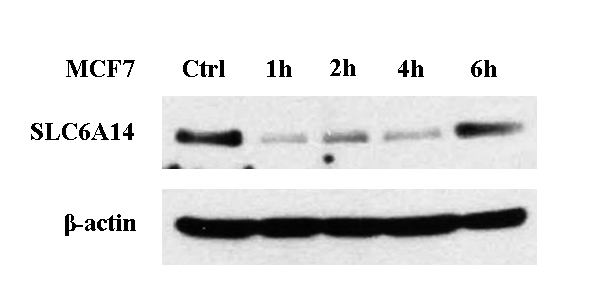


**Figure S3**. the ATB^0, +^ protein levels in MCF7 cells after treatment with LC-PLGA NPs for different time periods.


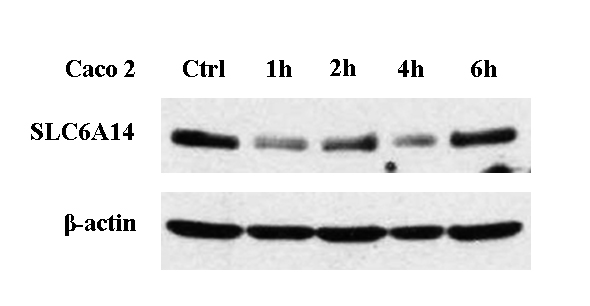


**Figure S4**. the ATB^0, +^ protein change in Caco-2 cells after treatment with LC-PLGA NPs for different time periods.


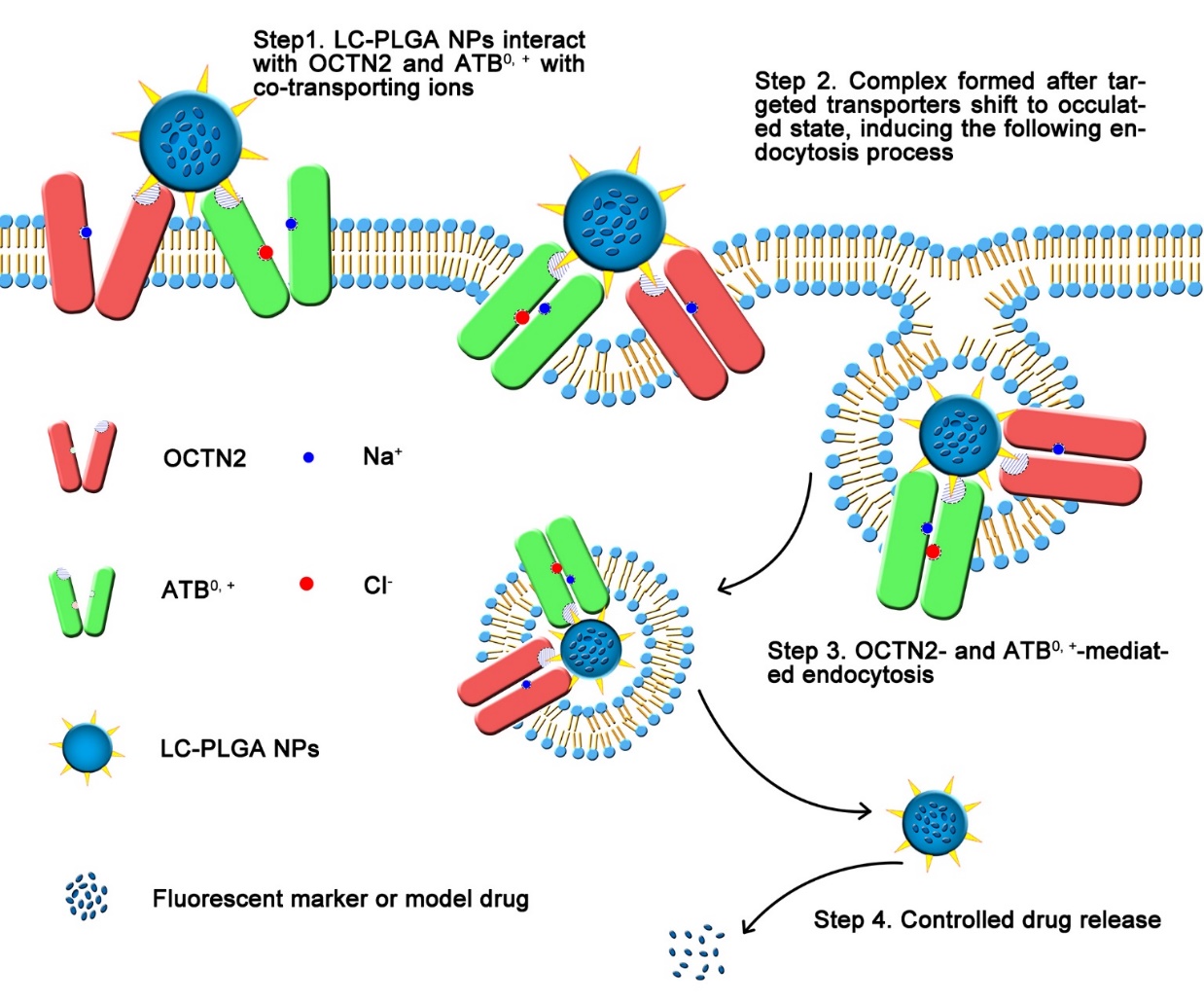


**Figure S5**. Graphical illustration of targeting LC-PLGA NPs targeting to both OCTN2 and ATB^0,+^. Firstly, in the presence of co-transporting ions, LC-PLGA NPs interact with OCTN2 and ATB^0,+^. The transporters shift from outward-facing conformation to occluded state, resulting in the formation of a complex, which then induces OCTN2- and ATB^0,+^-mediated endocytosis; after entry into cells, the encapsulated drug or marker was released from nanoparticles.


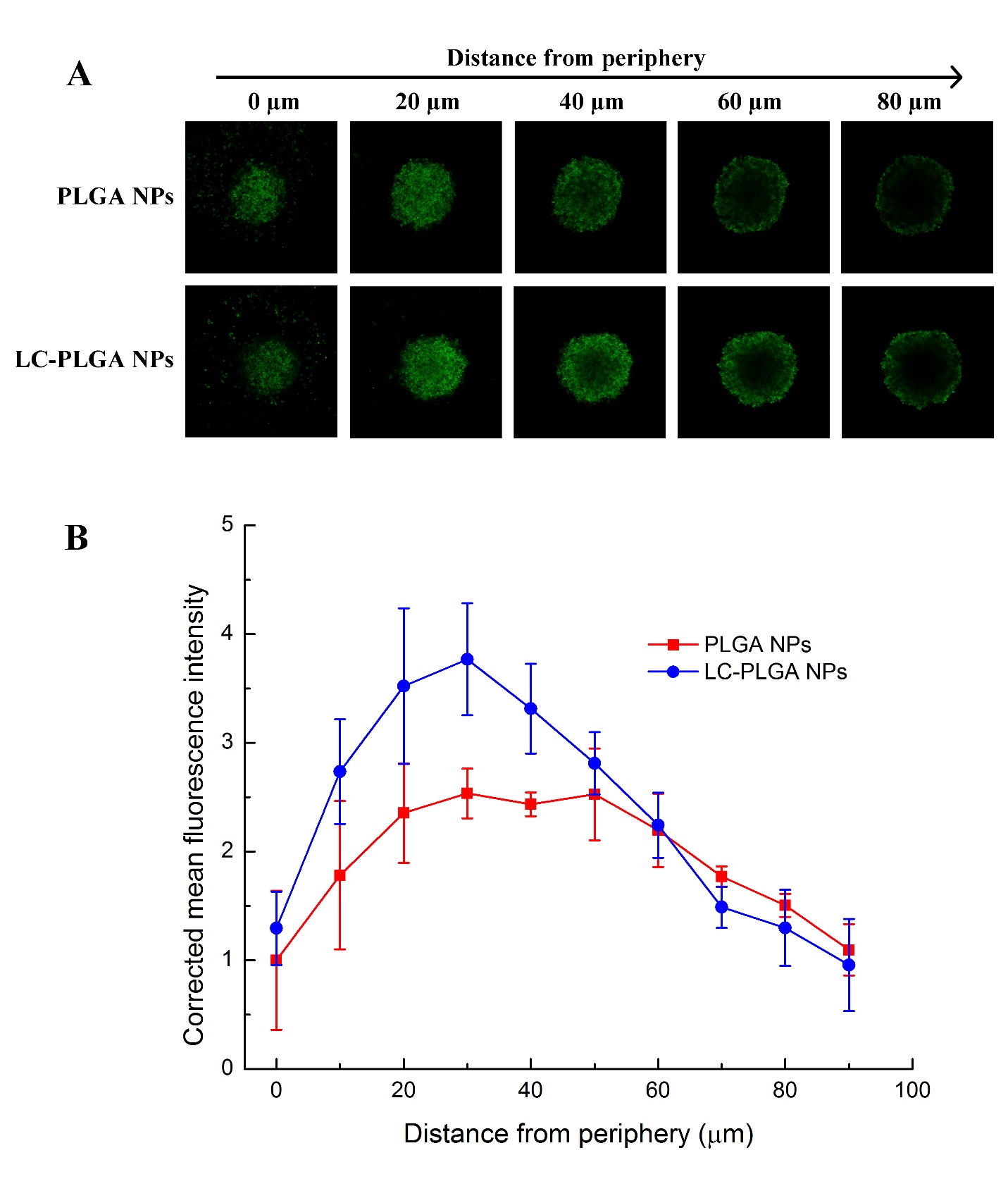


**Figure S6**. Nanoparticle penetration of spheroids. A, Z-stack images taken by confocal microscopy showing penetration of coumarin 6-labeled PLGA NPs and LC-PLGA NPs in HT29 spheroids. Green color indicates coumarin 6-labeled nanoparticles; B, Corrected coumarin 6 flourescence intensity represents the nanoparticles in spheroids from periphery to the inner layer (n = 3).


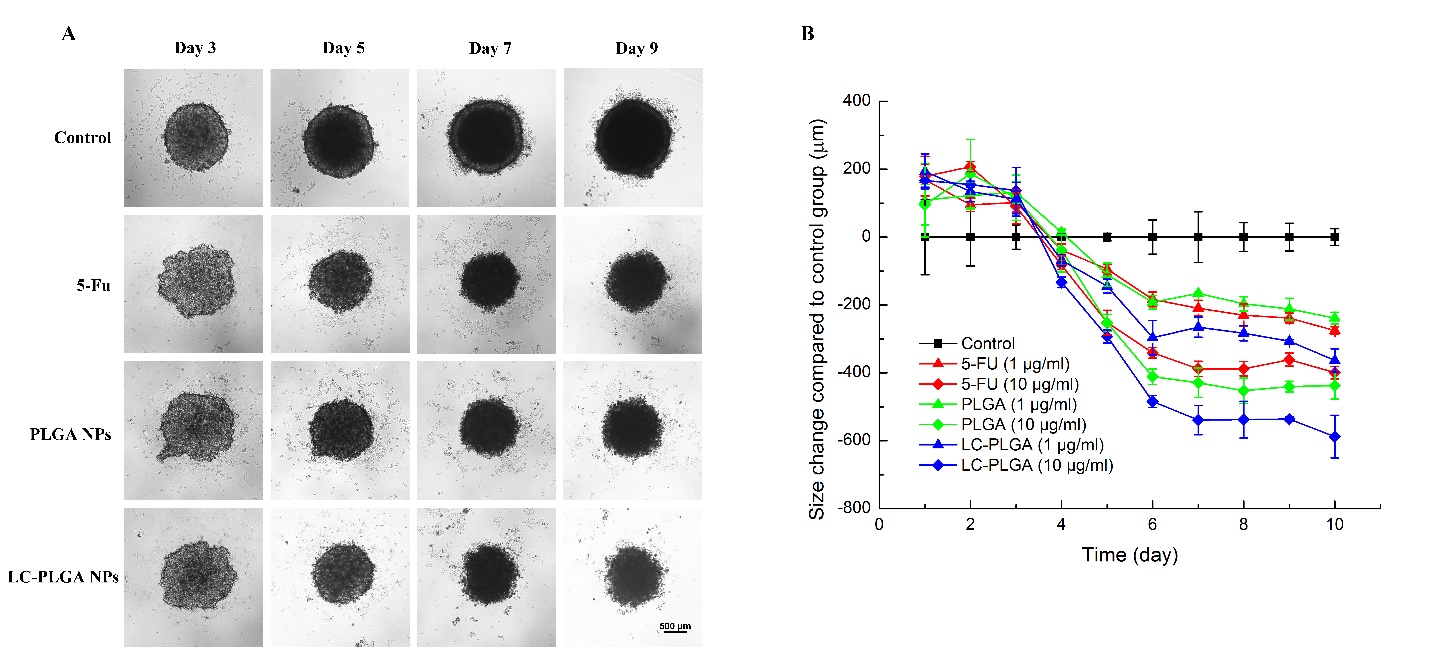


**Figure S7**. Spheroids treatment with free 5-FU, 5-FU-loaded PLGA NPs and 5-FU-loaded 10%LC-PLGA NPs. A, Morphological change in HT29 spheroids during 10-day treatment with 10 µg/mL of free 5-FU, 5-FU-loaded PLGA NPs and 5-FU-loaded 10%LC-PLGA NPs; B, Compared to control group, the size change of HT29 spheroids in response to treatment with 1 µg/mL and 10 µg/mL of free 5-FU, 5-FU-loaded PLGA NPs and 5-FU-loaded 10%LC-PLGA NPs (n = 3).
